# Clinical evaluation of pituitary insufficiency in adult population

**DOI:** 10.3906/sag-1908-19

**Published:** 2020-06-23

**Authors:** Selvihan BEYSEL, Mustafa ÇALIŞKAN, Muhammed KIZILGÜL, Seyfullah KAN, Mustafa ÖZBEK, Erman ÇAKAL

**Affiliations:** 1 Department of Endocrinology and Metabolism, University of Health Sciences,Dışkapı Yıldırım Beyazıt Training and Research Hospital, Ankara Turkey

**Keywords:** Pituitary insufficiency, pituitary adenoma, Sheehan’s syndrome

## Abstract

**Background/aim:**

This retrospective study aimed to investigate the clinical profile of pituitary insufficiency (PI) in adult population.

**Materials and methods:**

One hundred and fifty patients who were diagnosed as having PI between 2012 and 2018 (53.3% female, mean age 48.13 ± 15.83 years) were retrospectively analyzed.

**Results:**

Age at diagnosis was higher in females as compared with males (51.13 ± 15.95 vs. 44.70 ± 15.08 years, P = 0.012). The most frequent presenting signs were headache (29.4%) and visual disturbance (19.6%) in general. Females frequently presented with headache (33.3%), whereas males presented with sexual dysfunction (34.4%). The most frequent cause of PI was nonfunctional pituitary adenoma (28.8%) in general population. A frequent cause of PI was Sheehan’s syndrome (33.8%) among females and nonfunctional pituitary adenoma (38.6%) among males. Pituitary macroadenoma (75.8%) was frequent in pituitary tumors with PI. 55.3 % of the patients had 4 pituitary hormones deficiencies and 26.0% of patients had 3 pituitary hormones deficiencies. Gonadotropin deficiency was the leading pituitary hormone deficiency. The frequency of posttraumatic PI was 4.7% in the general population.

**Conclusion:**

Nonfunctional pituitary adenoma was the most common cause of PI among males and Sheehan’s syndrome was a major etiologic factor in females. Sheehan’s syndrome remains an important health problem in Turkey although obstetric care has improved. Posttraumatic PI should be considered in the differential diagnosis of idiopathic PI.

## 1. Introduction

Pituitary insufficiency (PI) in adults is usually acquired. The common causes of PI are pituitary adenoma and peripituitary tumors [1–3]. Other causes include pituitary apoplexy, postpartum pituitary necrosis [Sheehan’s syndrome (SS)], empty sella, stroke, traumatic brain injury (TBI), subarachnoid hemorrhage (SAH), and lymphocytic hypopituitarism [1–9]. Primary empty sella (PES) is defined as the thinning of the pituitary gland after subarachnoid space herniated into the sella turcica in patients with no history of pituitary tumor, radiotherapy, and surgery [4–6,10]. It occurs due to SS or lymphocytic hypophysitis [5,6]. Pituitary necrosis during delivery causes postpartum PI, which is referred as SS [11,12]. In a recent study, the estimated incidence for posttraumatic PI was more than 30 patients per 100.000 population [13]. Idiopathic PI is defined when no cause of PI can be found along with normal imaging studies [14]. The frequency of idiopathic PI was estimated as 8% [15]. A study from Turkey found that the most frequent cause of PI was pituitary tumors in males and nontumor causes in females [3]. This might be associated with delays in the diagnosis of PI because the initial symptoms are generally mild and nonspecific [1,16]. PI is associated increased mortality and morbidity, mainly due to the cardiovascular diseases caused by growth hormone (GH) deficiency [1,4,15–18]. Thus, the accurate early diagnosis of PI is important in long-term treatment and follow-up. 

Since PI’s features are not clearly reported in Turkish population, this study aimed to examine the clinical profile of pituitary dysfunction in patients with PI in Turkey. Clinical findings and hormonal results of patients with PI were retrospectively analyzed in this study. 

## 2. Materials and methods

One hundred and fifty patients with PI (53.3% female) who were treated in a tertiary referral endocrine center between 2012 and 2018 were enrolled in retrospective study. Patients aged over 18 years with at least 1 anterior pituitary hormone deficiency and/or the presence of diabetes insipidus was included. Mean age of the patients was 48.13 ± 15.83 (range, 19–83) years. Clinical findings including age at diagnosis, pituitary hormone profile, and etiology of PI were recorded. Subgroup analyses based on sex were performed to examine the clinical profile.

 PI was diagnosed using basal hormone concentrations and/or dynamic hormone tests. Serum free thyroxine, thyroid-stimulating hormone (TSH), prolactin, luteinizing hormone (LH), follicle-stimulating hormone (FSH), cortisol, growth hormone (GH), insulin-like growth factor 1, estradiol, testosterone concentrations were measured. Dynamic hormone tests including an insulin tolerance test, adrenocorticotrophic hormone (ACTH) stimulation test, gonadotropin-releasing hormone stimulation test, thyrotropin-releasing hormone stimulation test, and a water deprivation test were performed, as described in a previous study [12]. Panhypopituitarism was defined as three or more of the pituitary hormone deficiencies. Magnetic resonance imaging was performed in all subjects. Approval for the study was performed by the local bioethics committee of Dışkapı Training and Research Hospital (20.08.2015-25/05). Written consent was obtained from all participations. 

### 2.1. Statistical Analysis

All analyses were performed using the SPSS 18.0 (SPSS Inc., Chicago, IL, USA) statistics software package. Results are expressed as mean ± standard deviation (SD) or percentage (%). The chi-square test was used for the comparison of categorical variables between 2 groups. Student’s t-test was used to compare normally distributed continuous variables between 2 groups. The level of statistical significance was accepted as P < 0.05.

## 3. Results

Mean age at diagnosis was higher in females compared with males (51.13 ± 15.95 vs. 44.70 ± 15.08 years, P = 0.012). Clinical findings of patients with PI are shown in Table 1. The most frequent presenting sign was headache (29.4%), followed by visual disturbance (19.6%) in general. Females frequently presented with headache (33.3%), while males presented with sexual dysfunction (34.4%). The most common causes of PI were nonfunctional pituitary adenoma (28.0%), SS (18.0%), PES (16.0%), extrapituitary tumor (8.7%), idiopathic hypogonadotropic hypogonadism (5.3%), idiopathic PI (4.7%), and posttraumatic PI (4.7%), respectively, in general population. 

**Table 1:** Characteristics of pituitary insufficiency.

	Total(n = 150)	Female(n = 80)	Male(n = 70)	P*
Symptoms at diagnosis Headache Visual disturbance Nonspecific Sexual dysfunction Others** Secondary amenorrhea	29.4 19.6 17.6 16.7 10.8 5.9	33.3 11.1 27.8 - 16.7 11.1	25.0 29.2 6.2 35.4 4.2 -	0.001
Etiology Pituitary adenoma Nonfunctional adenoma Prolactinoma Cushing’s syndrome Acromegaly Gonadotropinoma Thyrotropinomas Sheehan’s syndrome Primary empty sella Extrapituitary tumors Idiopathic hypogonadotropic hypogonadism Idiopathic pituitary insufficiency Posttraumatic pituitary insufficiency Pituitary apoplexy	28.0 8.7 2.0 1.3 0.7 0.7 18.0 16.0 8.7 5.3 4.7 4.7 1.3	18.7 5.0 3.7 2.5 0.0 1.2 33.8 15.0 8.8 2.5 3.8 3.8 1.2	38.6 12.9 0.0 0.0 1.4 0.0 0.0 17.1 8.6 8.6 5.7 5.7 1.4	0.001

Others** included lack of lactation and galactorrhea. P-value* compares males vs. females

With regard to sex distribution, SS (33.8%), nonfunctional pituitary adenoma (18.7%), PES (15.0%), extrapituitary tumor (8.8%), idiopathic PI (3.8%), posttraumatic PI (3.8%), and idiopathic hypogonadotropic hypogonadism (2.5%) were common causes of PI among females. Nonfunctional pituitary adenoma (38.6%), PES (17.1%), extrapituitary tumor (8.6%), idiopathic PI (5.7%), posttraumatic PI (5.7%), and idiopathic hypogonadotropic hypogonadism (8.6%) were frequent causes of PI among men. Prolactinoma was major cause of the functional pituitary adenoma. Pituitary macroadenoma (75.8%) was frequent in pituitary tumors. Eighty-three patients (55.3 %) had 4 pituitary axes affected, 39 patients (26.0 %) had 3, 15 (10 %) had 2, and 13 patients (8.7 %) had 1 distributed pituitary axis. Gonadotropin deficiency (FSH/LH) was most common pituitary hormone deficiency (137 patients, 91.3%), followed by ACTH (125 patients, 83.3%), GH (110 patients, 73.3%), and TSH (117 patients, 78.0%) deficiencies. Posterior pituitary insufficiency was observed in 9 patients (6.0%). Age at diagnosis of pituitary insufficiency was higher in females with SS compared with those without SS (64.37 ± 9.01 vs. 44.38 ± 14.45 years, P < 0.01). Sex (female, 51.6% vs. 60.7%) and age (49.01±15.87 vs 44.32±15.33 year) was similar between patients with panhypopituitarism and those without panhypopituitarism (P > 0.05). We divided into 2 subgroups according to patients with panhypopituitarism (having 3 and 4 hormone deficiencies) and patients without panhypopituitarism (having 1 and 2 hormone deficiencies). Idiopathic was increased in patients without panhypopituitarism than in patients with panhypopituitarism (46.4% vs. 2.5 %, P < 0.001). Sheehan’s syndrome was increased in patients with panhypopituitarism than in patients without panhypopituitarism (21.3% vs. 3.6 %, P = 0.028). (Table 2) 

**Table 2:** The etiology of panhypopituitarism.

Etiology	Patients without panhypopituitarism (n = 28)	Patients with panhypopituitarism (n = 122)	P
Idiopathic* Pituitary adenoma without surgery Pituitary adenoma with surgery and/or radiotherapy Primary empty sella Apoplexy Sheehan syndrome*	46.4% 7.1% 28.6% 14.3% 0 3.6 %	2.5% 9.0% 44.3% 21.3% 1.6% 21.3%	< 0.001

*Idiopathic and Sheehan’s syndrome were significantly different (P < 0.05).

## 4. Discussion

This study showed that nonfunctional pituitary adenoma was common cause of PI among males and Sheehan’s syndrome was a major etiologic factor in females. Females frequently presented with headache while males presented with sexual dysfunction. Three and 4 pituitary hormone deficiencies were mostly observed. Gonadotropin deficiency was mostly presented. Posttraumatic PI was considered in diagnosis of PI.

The Society of Endocrinology and Metabolism of Turkey (SEMT) study found that the frequent cause of PI was pituitary tumors in males, whereas it was nontumor causes in females [3]. Nonfunctional pituitary adenoma, SS, PES, and extrapituitary tumors were represented as the frequent causes of PI in general population. According to sex, we showed that nonfunctional pituitary adenoma, PES, and extrapituitary tumors were the frequent causes of PI among males. Nonfunctional pituitary adenoma, SS, and PES were the frequent causes of PI among females.

The primary etiologic factor for PI in females was SS, whereas it was nonfunctional pituitary adenoma in males in our report. Nonfunctional pituitary adenoma was common cause of PI among general population as well as in males. The Spanish study in 2001 showed that the leading cause of PI was pituitary tumors. Idiopathic PI and SS were nontumoral causes of PI in Spanish population [2]. Pituitary tumors were found as the frequent cause of PI, and SS was rare cause of the PI in another study from Spain in 2013 [1]. 

Consistent with studies [1–3], the present report showed that nonfunctional pituitary adenomas were the leading cause for PI in general population. Percentage of nonfunctional pituitary adenomas was almost similar to a previous study performed in 2001 [2]. Nevertheless, there was a higher occurrence of nonfunctional pituitary adenoma in our report than in the SEMT study [3]. We showed that nonfunctional pituitary adenoma was mostly presented as macroadenoma (75.8%). Mass effect of tumor and extensive pituitary surgery might increase the prevalence of PI in nonfunctional adenomas. 

Our study showed that the second main cause was SS in general population. SS was also frequent cause among females. In developed countries, SS was responsible for 3.1% of PI, and PI was mostly caused by pituitary tumors [19]. In SEMT study, SS was found in 27.6% of females and 13.0% of the general population [3]. Gokalp et al. suggested that SS in Turkey was associated with postpartum bleeding [20]. This study showed that SS was the frequent cause in females. SS remains a major cause of PI among females in Turkey although obstetric care has improved. The greater improvement in obstetric care in developed countries is associated with the lower incidence of SS. This disorder remains the major cause of PI in developing world [11]. Previous studies have suggested that SS had more severe hormone deficiency than other causes of PI [11,12]. Panhypopituitarism was mostly observed in patients with SS [21], similar to our study. We showed that PES was the third frequent cause of PI in general population. Panhypopituitarism was mostly observed in patients with PES. Detailed endocrine assessment is needed for PES due to the increased percentage of anterior hormone deficiencies. 

We showed that idiopathic PI occurred in 4.7% of the general population and 5.7% of men. In a previous report, 8% of PI was found to be idiopathic [15]. After idiopathic PI with normal pituitary imaging was reexamined, these patients were actually diagnosed as having neurosarcoidosis, hemochromatosis, and cranial diabetes insipidus [14,22]. PROP-1, POU1F1, and HEX1 gene mutations were present in Turkish families with combined pituitary hormone deficiency [23,24]. PI after TBI, SAH, meningitis or stroke has also been reported [7–9].

The frequency of posttraumatic PI after TBI or SAH is more than 30 new cases per 100,000 population per year [13]. In our report, 4.7% of patients had secondary PI due to TBI, SAH, and stroke. Pituitary adenoma, congenital, head trauma, infections, and extrapituitary cranial irradiation compromised the higher frequency of the PI in Serbian population [25]. Similar to our findings, the prevalence of pituitary adenoma, mostly nonfunctional macroadenoma, in European Caucasian population were increased with the broader use of imaging techniques in routine practice [26]. Posttraumatic PI should be investigated especially in the differential diagnosis of idiopathic PI. The lower percentage of PI secondary to SAH or TBI in this report was possibly due to an inadequate awareness during the follow-up period. 

The frequent presenting symptom was headache and visual disturbance in our population, similar to previous findings [27]. Gonadotropin deficiency was predominantly pituitary hormone deficiency, consistent with previous study [3]. However, GH deficiency was most frequent in pituitary hormone deficiency [4,6,7,11,13,16,19,28].

The limitations of our study are that this was a retrospective study with a small sample size, and dynamic tests were not performed in all patients.

## 5. Conclusions

In conclusion,this study showed that the frequent cause of PI in general population was nonfunctional pituitary adenoma. Nonfunctional pituitary adenoma was mostly presented in males and SS was frequent in females. SS remains an important health problem in Turkey although obstetric care has improved. Posttraumatic PI should be considered in the differential diagnosis of idiopathic PI.

## Disclaimer/Conflict of interest 

All authors declare that they have no conflict of interest. The approval for the study was performed by the local bioethics committee of Dışkapı Training and Research Hospital (20.08.2015-25/05). 

## Informed consent

Written consent was obtained from all participants.
